# GEM System: automatic prototyping of cell-wide metabolic pathway models from genomes

**DOI:** 10.1186/1471-2105-7-168

**Published:** 2006-03-23

**Authors:** Kazuharu Arakawa, Yohei Yamada, Kosaku Shinoda, Yoichi Nakayama, Masaru Tomita

**Affiliations:** 1 Institute for Advanced Biosciences, Keio University, Fujisawa 252-8520, Japan

## Abstract

**Background:**

Successful realization of a "systems biology" approach to analyzing cells is a grand challenge for our understanding of life. However, current modeling approaches to cell simulation are labor-intensive, manual affairs, and therefore constitute a major bottleneck in the evolution of computational cell biology.

**Results:**

We developed the Genome-based Modeling (GEM) System for the purpose of automatically prototyping simulation models of cell-wide metabolic pathways from genome sequences and other public biological information. Models generated by the GEM System include an entire *Escherichia coli *metabolism model comprising 968 reactions of 1195 metabolites, achieving 100% coverage when compared with the KEGG database, 92.38% with the EcoCyc database, and 95.06% with iJR904 genome-scale model.

**Conclusion:**

The GEM System prototypes qualitative models to reduce the labor-intensive tasks required for systems biology research. Models of over 90 bacterial genomes are available at our web site.

## Background

Given the burgeoning wealth of knowledge in molecular biology, including the ever more rapidly accumulating quantitative high-throughput data, and with more than a hundred complete genomes now at hand, the grand challenge of what we might call "the post-genome era" is to obtain a system-level understanding of the dynamic behavior of the mechanisms of life. However, the dynamic behavior of biological systems, a result of the diverse nonlinear interactions of multiple molecular components possessing various properties, is complex and unintuitive. An integrative systems biology approach is therefore required to complement traditional reductionism, and computer simulation has proven to be an invaluable tool for system-level analysis [1]. Simulation-based research facilitates the understanding of the complex underlying structure of a system, and detailed models can be used to help generate testable predictions and hypotheses for experiments.

Several simulation studies of large-scale biological systems have been reported, but most are achieved by manual modeling of the cellular networks and simulation of network models by the use of approaches such as biochemical systems theory [2] and flux balance analysis [3]. Investigations of dynamic behavior thus far have been limited in scale, focusing on minimized models [4] or specific pathways [5]. This is mostly because dynamic modeling for biosimulation requires a multitude of parameters, and collection and organization of the information required is extremely time-consuming, labor-intensive, manually precise work. The modeling procedure usually involves three major steps: (1) qualitative modeling, where the network structure or the pathway map including all the necessary inhibitors, activators, reversibility, and feedback regulation is constructed from established biological knowledge and hypotheses; (2) quantitative modeling, where quantitative data such as the metabolite and enzyme concentrations, accurate rate equations, and kinetic parameters are incorporated so as to formulate a mathematical system model; and (3) cell programming, where the above information is translated into a machine-readable modeling language such as the Systems Biology Markup Language (SBML) [6] ready for simulation software, with specifications for simulation such as the type of integrators, integration step size, and simulation procedures [7]. The manual modeling process is the most serious bottleneck in systems biology, and an intelligent environment with both sophisticated data and knowledge bases is necessary for the next step in the evolution of computational cell biology. For example, the biochemical simulator GEPASI/COPASI [8] provides an intuitive graphical user interface to aid the cell programming process in modeling, yet the users are required to obtain the qualitative and quantitative information manually, and current biochemical simulation software suites do not provide the automatic qualitative and quantitative modeling components.

Although quantitative modeling currently requires a thorough bottom-up approach with expert knowledge and a large amount of public kinetic information, a substantial part of qualitative modeling can be automated by integrating information from numerous databases. Although not intended for generating simulation models, several software tools for the reconstruction of the pathway database from the genome exist, including metaSHARK [9], IdentiCS [10], and the PathoLogic program in the BioCyc Pathway Tools software suite [11]. However, PathoLogic, for example, heavily relies on text-based annotation of the genome, and sometimes requires another pipeline such as the GeneQuiz system for annotation beforehand [12]. Since all three software tools contain no stoichiometric information and lack the cell programming step, they all require considerable time and effort in order to use the results for simulation. In contrast, a system dedicated to the prototyping of pathway simulation models can be highly optimized for speed and ease of use.

Here we describe a novel database-driven intelligent software system named the Genome-based Modeling (GEM) System, which automatically generates a cell-wide metabolic pathway simulation model suitable as a draft model to build upon for computer-based studies. The model is based on complete genome sequence data, and the software provides an environment that allows analysis of the system-level behavior of the organism of interest.

## Results

### Approach

A traditional modeling approach scales up from a basic model by adding new information by hand, but because our aim is to provide a draft model to reduce the labor-intensive qualitative modeling steps, we take an opposite, top-down approach of automated modeling. A rough image of the entire metabolic network is extracted from genome data and modeled on the basis of genetic information, and then more specific information is later added from expert knowledge with minimal manual work. Another merit to this approach is that the process starts from the genome sequence. In whole-cell modeling, integration of different biological databases is a challenging task, because the target field is broad and the scheme of each database differs. Moreover, the names of genes and proteins are often ambiguous and thus difficult to match. However, most databases contain a link to the genome sequence regardless of the subject, so by modeling from the genome as the starting point, it becomes possible to link a large amount of biological information by an automated method.

### Qualitative modeling

**Figure 1:**
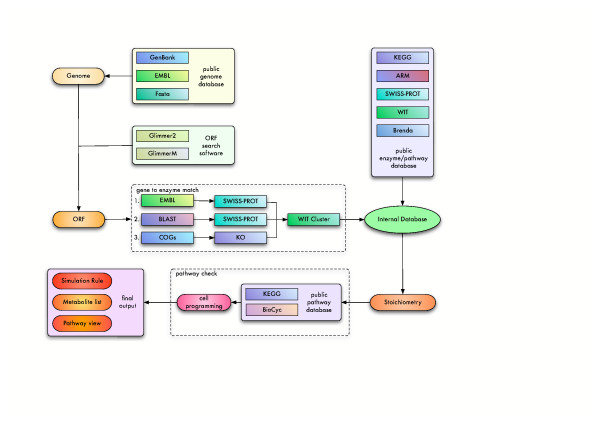
**The system workflow. **Starting from a genome sequence, all coding regions are matched to corresponding reaction stoichiometry for qualitative modeling, and then the reactions are quantitatively modeled with kinetic equations to generate a cell-wide simulation model.

The GEM System takes the complete genome database, both annotated and unannotated data, as input, and automatically goes through several steps to produce a simulation model of the organism in a flat-file format that can be readily converted to standard SBML suitable for simulation with various simulation software systems (Figure 1) [7,13]. When an unannotated genome is given, the system predicts genes with Glimmer [14], which produces a high false-positive rate and a low false-negative rate. Alternatively, users may use existing sophisticated pipelines such as GenDB [15], GeneQuiz [16], and EnsEMBL [17] to identify the coding regions and for the functional annotation to be used in the following steps.

The second step matches the genes to the product protein through a combined method of annotation reference, homology search, and orthology search. The system first checks for a direct external database link (db_xref) to SWISS-PROT and TrEMBL [18] in the annotation frequently provided in the EMBL complete genome database [19] to find the EC number of the protein product of a gene. If annotation is not available, the system performs a BLASTP search [20] against the SWISS-PROT database with a default cutoff value of e-25, which is also configurable, and the SWISS-PROT entry with the best e-value is used as its homolog. Homology searching is a powerful technique for conserved proteins, but sometimes is insufficient in functional genomics [21]. If there is no hit above the cutoff e-value, an orthology search of the amino acid sequence of the gene by using the COGnitor program provided with the COGs database [22] is then performed with a cut-off value at 3 clades. This step can alternatively be carried out by direct reference to the annotated COG entry given in the PTT database distributed in the GenBank genome flat-file [23]. The obtained COG entry is matched to the corresponding EC number by reference to the KEGG Orthology database [24].

When the SWISS-PROT and TrEMBL entries match with the annotation and the homology search does not contain an EC number, there is an additional search in the ortholog entries in the same WIT Cluster [25] category. The WIT Cluster provides a list of orthologous genes identified under strict criteria; therefore, the genes in the same WIT Cluster are expected to have identical functions. Because the GEM System keeps track of the enzymes by the EC number, when the SWISS-PROT and TrEMBL entries are not annotated with an EC number, the system looks through all orthologous genes in the same WIT Cluster to find entries annotated with an EC number, and uses those for annotation. When multiple entries with EC numbers are found, the entry with the lowest e-value is used. Currently, the GEM System cannot account for any enzymes that are not EC-encoded, and unspecific or incomplete codes are treated the same as complete codes. This is a limitation of the system, but since the KEGG database that the system uses to check the pathway is also mostly based on EC numbers and treats unspecific or incomplete codes similarly, our system follows this approach.

The GEM System holds information on enzymes extracted from the major enzyme and pathway databases [18,24-27] and curates it for consistency of nomenclature in the form of an internalized database, and the EC numbers obtained are matched to the corresponding stoichiometric enzyme reaction equation. Here each gene is assumed to have a one-to-one enzyme relationship, so the system cannot distinguish between isozymes and heteropolymeric enzymes. To resolve this problem and to recover false negative matches, the stoichiometric reaction list undergoes a pathway check that compares the extracted list with the general reference pathway of the KEGG and MetaCyc databases [28]. For the problem of many-to-many relationships between reactions, firstly the multiple rows with the same stoichiometry (the reactions are equivalent in each instance) are collapsed to a single row. This procedure is equivalent to resolving the problem of heteropolymeric enzymes and ignoring the presence of isozymes. With this new stoichiometric matrix, where all rows represent unique reactions, each reaction is searched in the reference pathways, and the row is duplicated when the reaction exists in multiple pathways, to recover the necessary isozymes. Then for the pathway connectivity check, when fewer than "Y" steps of a gap exist between more than "X" connected steps upstream and downstream of the pathway, the gap is filled from the information in the pathway databases. X and Y are configurable, but are set to 3 and 1, respectively, by default. For example, when there is a continuous pathway containing 7 enzymes such as A-B-C-D-E-F-G exist, and when the consecutive sets of three enzymes A-B-C and E-F-G are identified by functional annotation, the gap enzyme D is filled in. All 7 enzymes in this example must be consecutive reactions, but they are not required to be in linear order or to belong in the same pathway. Gap filling in pathway reconstruction is a challenging task, and the filled gap would be better to be reconfirmed by sequence alignment. Although only the most straightforward method is currently implemented, the filled gap is clearly marked as such in the model, therefore enabling the users to easily take out uncertain reactions. This gap-filling process is useful for flux-based analysis where pathway connectivity is essential, but since the data is uncertain, these reactions are not included for the following validations. A graphical user interface allows the configuration of options and optimal execution of the system.

To summarize the procedures described so far, let us follow the workflow taking fumarate hydratase (4.2.1.2) and glutamate synthase (1.4.1.13) in *E. coli *as examples. Firstly, the coding regions are identified by Glimmer unless an annotated complete genome is available, and the nucleotide sequences are translated into amino acid sequences. If database reference to SWISS-PROT is available in annotation, this reference is used to identify the gene and the EC number of enzyme coded by the gene, and if the reference is not available, BLAST similarity search matches the amino acid sequence to the corresponding SWISS-PROT entry. In this case, gene located on the complementary strand of position 1684755 to 1686401 is identified to be FUMA_ECOLI that is 4.2.1.2 in EC number with complete identify (e-value of zero), and likewise, FUMB_ECOLI (e-value of zero) is also identified to be 4.2.1.2. Similarly, two genes GLTB_ECOLI (e-value of zero) and GLTD_ECOLI (e-value of 6.5e-282) are identified to be 1.4.1.13. Even when the homology search fails, orthology search identifies *fumA *and *fumB *genes to be both COG1838 (Tartrate dehydratase beta subunit/Fumarate hydratase class I, C-terminal domain), and the majority of genes belonging to the orthologous cluster is identified to be 4.2.1.2 in WIT Cluster. Likewise, *gltB *is identified to be COG0069 (Glutamate synthase domain 2) and *gltD *to be COG0493 (NADPH-dependent glutamate synthase beta chain and related oxidoreductases) and subsequently to be 1.4.1.13 with WIT Cluster. FumA is an aerobic isozyme and FumB is an anaerobic isozyme, whereas GltB and GltD are subunits of glutamate synthase. This is correctly identified by pathway check, because 4.2.1.2 occurs both in aerobic citrate cycle and in anaerobic reductive carboxylate cycle pathways, but 1.4.1.13 only occurs in Glutamate metabolism pathway (1.4.1.13 actually also exists in nitrogen metabolism pathway in the GEM model, but this is from another isozyme, *gltF*). Reaction for 4.2.1.2 is a reversible reaction of (S)-malate = fumerate + H2O, and that of 1.4.1.13 is an irreversible reaction of 2 L-glutamate + NADP = L-glutamate + 2-oxoglutarate + NADPH + H. Taking account of the existence of isozymes (here we igonore *gltF *for convenience), the stoichiometric matrix is derived as follows (Table 1):

where the first four rows represent two sets of reversible reaction of 4.2.1.2 and the fifth row represents the irreversible reaction of 1.4.1.13, and the columns represent (S)-malate, fumerate, H2O, L-glutamate, NADP, 2-oxoglutarate, NADPH, and H, respectively. For gap-filling, the reaction connecting 2-Hydroxy-ethyl-ThPP and Acetaldehyde, namely 4.1.1.1 is suggested from the connectivity of upstream reactions (4.2.1.11, 2.7.1.40, and 1.2.4.1) and downstream reactions (1.2.1.3, 6.2.1.1, and 2.3.1.12) in the glycolysis pathway.

### Validation of the qualitative modeling step

Stoichiometric simulation models of all available complete annotated bacterial genomes have been generated by using the GEM System with the default parameters. Complete genome flatfiles were obtained from the EMBL database, and corresponding PTT files were used for COG annotation. Using these inputs, the BLAST searches were limited to the genes that did not contain direct external database link (db_xref) to SWISS-PROT or TrEMBL, and COG searches were replaced with data integration of PTT file with EMBL data. In this way, functional annotation process was optimized and therefore the calculation speed was remarkably fast, finishing the entire process in a few hours on a dual-processor PC server (Pentium 4 Xeon 2.8 GHz, 4 GB RAM). Statistics describing the scale of computer-based cell models with over 500 enzymes are shown in Table 2 (the complete list of 90 models is available at our web site [29]). Here the KEGG coverage is calculated as the percentage of correctly extracted enzymes in the corresponding organism-specific pathways in the KEGG database. The model organism *E. coli *K12 MG1655 yielded the best numbers, with 968 reactions of 835 enzymes and 1195 metabolites in the computer-based model with an accuracy of 100% KEGG coverage, in other words, without any false negatives. Organisms that are not well understood are limited by this database driven approach, but even the model with lowest coverage, in this case *Mycobacterium leprae*, achieved over 90% coverage.

Accuracy comparison with *E. coli* specific entries of KEGG PATHWAY and SWISS-PROT data for pathways with unidentified genes or genes that have no EC number assigned are shown in Table 3. Since the overall KEGG coverage is 100% in *E. coli*, the EC coverage is obviously 100% for all pathways. However, there are several reactions that cannot be EC coded in some metabolic pathways, and a large fraction of reactions for pathways other than metabolism. Although the reactions that are not EC coded is not included in the stoichiometric model since they are beyond the purpose of this work to create metabolic pathway models, GEM System correctly identified all genes except for 3 cases in comparison with SWISS-PROT entries, so that the information can easily be incorporated for future applications. Three misidentified genes were actually correctly identified but the homology search identified them in different organisms or strains, namely, GPMA_ECOLI was identified to be GPMA_SHIFL (same gene in *Shigella flexneri*), ISPH_ECOLI and UPK_ECOLI were identified to be ISPH_ECO57 and UPPP_ECO57 (same gene in O157 strain of *E. coli*).

The *E. coli *model was further compared with the genome-scale metabolic flux model of Reed *et al*. (iJR904) [30] and the EcoCyc database [31]. EC numbers are directly compared, and all of the 54 enzymes that are not included in the *E. coli *specific entries of KEGG or SWISS-PROT are manually checked through EcoCyc and iJR904 as shown in Table 4. There were 6 possible mis-annotations by the GEM System, but the majority of the enzymes were mis-identified due to the inconsistencies of the EC notation among databases. After correction of obsolete or deleted EC numbers, iJR904 contained 388 out of 425 EC numbers in common (91.29% accuracy), and EcoCyc had 651 out of 701 EC numbers in common (92.38% accuracy). 16 enzymes that were assigned different EC numbers between the SWISS-PROT database and the iJR904 model, although the genes were correctly identified, so our model has overall 95.06% accuracy compared with the iJR904 model. Five enzymes out of the 21 false negatives in comparison with iJR904 and 38 out of the 49 false negatives in comparison with EcoCyc have no corresponding genes as of now. This fact emphasizes the importance of manual refinement, but since this process is required for less than 5% of the model in *E. coli*, our automatic modeling system should keep the manual effort to a minimum. Obviously *E. coli *is the most well studied organism, and the manual procedure required for other organisms would be greater than 5%. However, most of the other models also yielded over 500 reactions at 90% or more KEGG coverage, and since the models are provided with pathway-wise accuracy table similar to Table 3 at GEM System web-site [29] the user can easily identify which pathway is incomplete and thus requires manual checking.

The number of EC numbers extracted from the iJR904 model, 425, may seem small compared with the total number of reactions, 931. However, iJR904 contains 184 transporters that cannot be EC-coded, and it contains many enzymes without EC numbers that are EC-coded in KEGG database. Moreover, since many enzymes have multiple reactions, the total number of EC-coded reactions in iJR904 is 519. It is worth noting that iJR904 selects the pathways to include in the model, whereas the GEM System takes a greedy approach where every possible enzyme that is predicted to exist in a genome is included, regardless of the types of pathway the enzyme belongs to, leading to greater number of enzymes than in the iJR904 model. To summarize, the generated model has very high coverage (91~100%) compared to KEGG, EcoCyc, and iJR904, and the overall accuracy is also high, with false-positives of 6 entries (0.72%) and possible false-negatives of less than 43 entries (5.14%).

### Database of generated models

Our web site [29] makes publicly available all genome-scale models with enzyme or metabolite lists with reactions, gene lists with matched product and BLAST e-values, stoichiometric matrices for static simulation and metabolic flux analysis, interactive pathway maps generated with a Java applet for visualizing protein-protein interactions [32], and a tool to view the extracted enzymes mapped on the KEGG pathway database by using KEGG API.

## Discussion

We have developed the GEM System, automated software for the rapid construction of draft simulation models of cell-wide metabolic pathways from genome sequence information by integration of public biological databases. Automatic generation of the models is currently limited to metabolism in bacteria, and depends on the availability of EC numbers in public databases, but we have shown that qualitative models of the metabolic pathways of bacteria can be generated with low false positives and negatives, as validated by the comparison with KEGG, EcoCyc, and Reed *et al*.'s model. Although the generated models are draft models and thus still require expert curation to ensure the accuracy of simulations, manual involvement is minimized.

There are, however, several limitations of this approach. Firstly, although EC numbers are generally effective for enzyme data representation for well known pathways, certain number of reactions have no EC number assigned, and therefore majority of the transporters are identified as genes but not included as reactions in GEM System, making a large fraction of the model different from iJR904. Secondly, some EC numbers are incomplete and therefore ambiguous, and some become quickly obsolete, being assigned to new EC numbers. This resulted in more than 40 inconsistent enzyme assignments in GEM System. Thirdly, since the GEM System identifies enzymes and the corresponding reactions based on the genome information, it cannot identify reactions that are experimentally observed but with no corresponding gene found. To overcome these problems, more general nomenclature for enzymes should be used in addition to the EC numbers and integrate necessary information that have no link to the gene sequences.

The system generates a stoichiometric simulation model in SBML format, which is readily applicable to flux-based analyses on a number of simulation platforms. The stoichiometric models can be used for metabolic flux analyses by supplying experimental data for exchange fluxes as reported elsewhere [33,34]. One potential application of GEM System using this stoichiometric matrix is for dynamic large-scale simulation of metabolic pathways with hybrid dynamic/static simulation method [35]. Using this method, quasi-dynamic simulation is achieved by subdividing the model into multiple "static modules" connected by "dynamic modules", and by calculating the flux distribution of static modules using the stoichiometry and boundary flux of the dynamic module that is modeled with traditional enzyme kinetics methods. In this way, necessary kinetic equations and parameters are significantly reduced while maintaining simulation accuracy. Most reactions with high elasticities can be included in the static module, for which the stoichiometric matrix generated by the GEM System is directly applicable.

The GEM System can generate models automatically from public databases, but can also utilize private databases if such experimental data becomes available. Mining of high-throughput data by bioinformatics may facilitate the quantitative modeling step; for example, it should be possible to take advantage of recent progress in "metabolomics". Once genome-wide metabolome data becomes available via high-throughput techniques such as the capillary electrophoresis – electrospray ionization – mass spectrometry (CE-ESI-MS) method, metabolome data can be used to add unknown pathways, to supply the initial values of the metabolites, and to optimize kinetic parameters. Parameter fitting of time-series metabolite concentration data to general dynamic equations such as Generalized Mass Action is a possible substitution for accurate kinetic modeling, at least in the given time frame of the data set used for parameter optimization.

Our next step is to model the gene expression layer, including transcription, translation, and degradation processes. The GEM System is a powerful platform for this purpose, in no small part because the genome-based approach enables a link to databases of different fields based on the nucleotide sequences already described. Because the GEM System has been based on a generic bioinformatics workbench, that is, the G-language Genome Analysis Environment [36], the system can directly access genome sequences and perform computational genome data-mining. Required parameters or information such as the structure of a promoter can be directly obtained from the genome sequence as the simulation takes place. In this respect, GEM System can be extended to be applicable for the modeling of eukaryotes, by identifying protein subcellular localizations from database reference and with predictable methods [37,38]. Although the parameters in the functional annotation process should be revised to cope with the information availability and the existence of a multitude of duplicate gene paralogs, by selecting tissue specific gene expression pattern with expressed sequence tags (EST) or microarray data, the general approach of the GEM System should also be applicable for tissue specific cellular models of higher eukaryotes. In sum, the rapid accumulation of biological information now allows the realization of integrative systems biology, but at the same time makes manual modeling unrealistic; therefore, a genome-based automatic modeling procedure is a crucial step forward for the grand challenge to construct life in a computer.

## Conclusion

The GEM System facilitates systems biology research by prototyping a metabolic pathway simulation model from a genome. Given a complete genome, all modeling procedures are automated with configurable options, generating stoichiometric models in SBML format that are readily usable by cell simulators. In comparison with the KEGG organism-specific databases, the qualitative modeling step has high accuracy, with few false positives and negatives. More than 90 models generated from complete bacterial genomes are available for download online, with visualized pathway maps and gene lists.

## Authors' contributions

KA conceived the system, developed the software, carried out the validations, and drafted the manuscript. YY and KS participated in the design of the software. YN and MT supervised the work. All authors read and approved the final manuscript.

## Figures and Tables

**Table 1:** Stoichiometric matrix derived from the example procedure.

(1−1−100000−111000001−1−100000−1110000000011−1−1−1) MathType@MTEF@5@5@+=feaafiart1ev1aaatCvAUfKttLearuWrP9MDH5MBPbIqV92AaeXatLxBI9gBaebbnrfifHhDYfgasaacH8akY=wiFfYdH8Gipec8Eeeu0xXdbba9frFj0=OqFfea0dXdd9vqai=hGuQ8kuc9pgc9s8qqaq=dirpe0xb9q8qiLsFr0=vr0=vr0dc8meaabaqaciaacaGaaeqabaqabeGadaaakeaadaqadaqaauaabaqafGaaaaaaaeaacqaIXaqmaeaacqGHsislcqaIXaqmaeaacqGHsislcqaIXaqmaeaacqaIWaamaeaacqaIWaamaeaacqaIWaamaeaacqaIWaamaeaacqaIWaamaeaacqGHsislcqaIXaqmaeaacqaIXaqmaeaacqaIXaqmaeaacqaIWaamaeaacqaIWaamaeaacqaIWaamaeaacqaIWaamaeaacqaIWaamaeaacqaIXaqmaeaacqGHsislcqaIXaqmaeaacqGHsislcqaIXaqmaeaacqaIWaamaeaacqaIWaamaeaacqaIWaamaeaacqaIWaamaeaacqaIWaamaeaacqGHsislcqaIXaqmaeaacqaIXaqmaeaacqaIXaqmaeaacqaIWaamaeaacqaIWaamaeaacqaIWaamaeaacqaIWaamaeaacqaIWaamaeaacqaIWaamaeaacqaIWaamaeaacqaIWaamaeaacqaIXaqmaeaacqaIXaqmaeaacqGHsislcqaIXaqmaeaacqGHsislcqaIXaqmaeaacqGHsislcqaIXaqmaaaacaGLOaGaayzkaaaaaa@5C18@

**Table 2:** Generated bacterial pathway models. Bacterial models generated with GEM System from the complete genomes containing more than 500 enzymes is listed here (complete list of 90 models is available at our web site [29]). Here the KEGG coverage is calculated as the percentage of correctly extracted enzyme in the corresponding organism specific pathways in the KEGG database.

species	genes	metabolites	reactions	enzymes	KEGG coverage
*Bacillus anthracis Ames*	5311	1007	776	675	423/ 457(92.56%)
*Bordetella bronchiseptica RB50*	4994	1038	788	669	429/ 460(93.26%)
*Bacillus halodurans C-125*	4066	1056	812	693	422/ 435(97.01%)
*Bradyrhizobium japonicum USDA110*	8317	1257	995	863	521/ 555(93.87%)
*Bordetella parapertussis 12822*	4185	1011	763	644	404/ 433(93.30%)
*Bordetella pertussis Tohama I*	3447	957	717	602	394/ 422(93.36%)
*Bacillus subtilis 168*	4106	1060	818	699	464/ 476(97.48%)
*Bacteroides thetaiotaomicron VPI-5482*	4778	912	696	583	361/ 379(95.25%)
*Caulobacter crescentus CB15*	3737	1068	807	689	405/ 420(96.43%)
*Corynebacterium efficiens YS-314*	2950	944	674	574	352/ 383(91.91%)
*Chromobacterium violaceum ATCC 12472*	4407	1047	834	729	484/ 523(92.54%)
*Escherichia coli CFT073*	5379	1120	906	780	525/ 554(94.77%)
*Escherichia coli O157:H7 EDL933*	5349	1197	972	842	545/ 566(96.29%)
*Escherichia coli K-12 MG1655*	4289	1195	968	835	579/ 579(100.00%)
*Escherichia coli O157:H7 Sakai*	5361	1206	972	842	548/ 568(96.48%)
*Fusobacterium nucleatum ATCC 25586*	2067	804	608	514	302/ 326(92.64%)
*Gloeobacter violaceus PCC7421*	4430	945	689	587	347/ 375(92.53%)
*Haemophilus influenzae Rd KW20*	1709	907	664	551	358/ 359(99.72%)
*Lactococcus lactis subsp. lactis IL1403*	2266	925	683	567	312/ 328(95.12%)
*Mycobacterium bovis subsp. bovis AF2122/97*	3920	943	690	592	414/ 456(90.79%)
*Mycobacterium leprae TN*	1605	851	605	501	285/ 315(90.48%)
*Mycobacterium tuberculosis CDC1551*	4187	1064	780	678	403/ 424(95.05%)
*Mycobacterium tuberculosis H37Rv*	3869	1070	806	684	405/ 431(93.97%)
*Nitrosomonas europaea ATCC 19718*	2461	832	621	514	334/ 360(92.78%)
*Neisseria meningitidis Z2491 (serogroup A)*	2065	895	663	562	334/ 346(96.53%)
*Neisseria meningitidis MC58 (serogroup B)*	2025	867	635	532	329/ 339(97.05%)
*Oceanobacillus iheyensis HTE831*	3496	967	731	617	398/ 435(91.49%)
*Pseudomonas aeruginosa PA01*	5565	1218	944	826	499/ 517(96.52%)
*Photorhabdus luminescens*	4683	1026	785	672	458/ 501(91.42%)
*Pasteurella multocida PM70*	2014	913	671	561	372/ 391(95.14%)
*Pseudomonas putida KT2440*	5350	1087	840	722	456/ 492(92.68%)
*Pseudomonas syringae pv. tomato DC3000*	5471	1077	841	719	446/ 483(92.34%)
*Rhodopirellula baltica (Pirellula sp. strain 1)*	7325	971	748	639	412/ 432(95.37%)
*Staphylococcus aureus MW2*	2632	866	634	541	343/ 360(95.28%)
*Staphylococcus epidermidis ATCC 12228*	2419	843	612	514	328/ 350(93.71%)
*Shigella flexneri 301 (serotype 2a)*	4180	1084	858	736	493/ 532(92.67%)
*Shigella flexneri 2457T (serotype 2a)*	4068	1044	817	701	486/ 533(91.18%)
*Salmonella typhi Ty2*	4323	1100	887	764	527/ 563(93.61%)
*Synechococcus sp. WH 8102*	2517	804	575	508	347/ 375(92.53%)
*Thermosynechococcus elongatus BP-1*	2475	851	628	512	318/ 350(90.86%)
*Thermotoga maritima MSB8*	1846	862	622	515	292/ 305(95.74%)
*Xanthomonas axonopodis pv. citri 306*	4312	1054	804	679	424/ 451(94.01%)
*Xanthomonas campestris pv. ATCC 33913*	4181	1051	809	685	437/ 462(94.59%)
*Yersinia pestis KIM*	4090	1075	846	723	472/498(94.78%)

**Table 3:** Validation of *E. coli *model with KEGG and SWISS-PROT. Generated pathway model of *E. coli *was validated with *E. coli *specific entries of KEGG PATHWAY and SWISS-PROT database for every pathway. GEM System maintained high accuracy for proteins that are not EC coded. Three genes that are not identified actually correctly identified the gene, but in different organisms or strains. Similar table is available for all other models at the web database [29].

**Pathway**	**EC coverage**	**Gene coverage**	**Genes without EC **	**Unidentified genes**
00010:Glycolysis/Gluconeogenesis	26/26 (100%)	43/44 (97%)	1	GPMA_ECOLI
00051:Fructose and mannose	26/26 (100%)	53/53 (100%)	3	
00052:Galactose metabolism	17/17 (100%)	28/28 (100%)	2	
00061:Fatty acid biosynthesis (path 1)	7/7 (100%)	11/11 (100%)	1	
00100:Biosynthesis of steroids	10/10 (100%)	9/10 (90%)	0	ISPH_ECOLI
00130:Ubiquinone biosynthesis	12/12 (100%)	32/32 (100%)	1	
00190:Oxidative phosphorylation	8/8 (100%)	41/41 (100%)	1	
00511:N-Glycan degradation	2/2 (100%)	5/5 (100%)	1	
00550:Peptidoglycan biosynthesis	12/12 (100%)	16/17 (94%)	0	UPK_ECOLI
00620:Pyruvate metabolism	33/33 (100%)	45/45 (100%)	1	
00640:Propanoate metabolism	18/18 (100%)	24/24 (100%)	1	
00750:Vitamin B6 metabolism	9/9 (100%)	11/11 (100%)	1	
00760:Nicotinate and nicotina	16/16 (100%)	16/16 (100%)	1	
02010:ABC transporters prokaryotic	4/4 (100%)	190/190 (100%)	186	
02020:Two-component system	11/11 (100%)	85/85 (100%)	38	
02030:Bacterial chemotaxis	3/3 (100%)	20/20 (100%)	17	
02040:Flagellar assembly	1/1 (100%)	38/38 (100%)	37	
02060:Phosphotransferase sys	2/2 (100%)	53/53 (100%)	13	
03010:Ribosome	0/0 (%)	55/55 (100%)	55	
03030:DNA polymerase	1/1 (100%)	13/13 (100%)	1	
03060:Protein export	2/2 (100%)	17/17 (100%)	15	
03070:Type III secretion system	1/1 (100%)	10/10 (100%)	9	
03090:Type II secretion system	2/2 (100%)	25/25 (100%)	23	

**Table 4:** Check for all 54 enzymes not found in KEGG or SWISS-PROT. All of the 54 enzymes that were not found in *E. coli *specific entries of KEGG PATHWAY or SWISS-PROT database were manually checked with EcoCyc and iJR904. Although there were 6 probable mis-annotations by the GEM System, most enzymes were correctly identified in EcoCyc. This is mostly due to the inconsistencies of EC numbers among databases.

**GEM**	**gene name**	**EcoCyc**	**KEGG(ECOLI)**	**Swissprot(ECOLI)**	**iJR904**	**description**
1.10.2.-	cyoA-D, appB-C	1.10.2.-	1.10.3.-	1.10.3.-	no EC	inconsistency of EC (incomplete EC)
1.14.3.-	ubiH	1.14.3.-	1.14.13.-	1.14.13.-	N.A.	inconsistency of EC (incomplete EC)
1.16.1.-	ndh	1.16.1.-	1.6.99.3	1.6.99.3	N.A.	inconsistency of EC (incomplete EC)
1.17.4.-	nrdA-B, E-F	1.17.4.-, 1.17.4.1	1.17.4.1	1.17.4.1	N.A.	inconsistency of EC (incomplete EC)
1.18.99.1	hyfA-F, H	no EC	1.-.-.-	1.-.-.-	N.A.	possible misannotation by GEM System (EC not applicable)
1.2.1.19	feaB	1.2.1.39	1.2.1.39	1.2.1.39	1.2.1.39	missannotation by GEM System
1.2.1.21	aldA	1.2.1.21	1.2.1.21, 1.2.1.22	1.2.1.21, 1.2.1.22	1.2.1.21	KEGG PATHWAY mainly uses 1.2.1.22
1.2.1.24	feaB	1.2.1.39	1.2.1.39	1.2.1.39	1.2.1.39	missannotation by GEM System
1.3.-.-	frdD	1.3.5.-	1.3.99.1	N.A.	1.3.99.1	inconsistency of EC (incomplete EC)
1.3.1.10	fabI	1.3.1.9, 1.3.1.10	1.3.1.9	1.3.1.9	N.A.	1.3.1.10 specifically functions as 1.3.1.9 in E.coli
1.4.3.-	nadB	1.4.3.-	1.4.3.16	1.4.3.16	N.A.	inconsistency of EC (incomplete EC)
1.5.3.2	solA	1.5.3.2	1.5.3.1	1.5.3.-	N.A.	inconsistency of EC
1.6.4.2	gor	1.8.1.7	1.8.1.7	1.8.1.7	N.A.	inconsistency of EC (1.6.4.2 is changed to 1.8.1.7)
1.6.4.5	trxB	1.6.4.5	1.8.1.9	1.8.1.9	1.6.4.5	inconsistency of EC (1.6.4.5 is changed to 1.8.1.9)
1.6.6.-	nirB	1.7.1.4	1.7.1.4	1.7.1.4	no EC	inconsistency of EC (1.6.6.4 is changed to 1.7.1.4)
1.6.6.8	guaC	1.6.6.8	1.7.1.7	1.7.1.7	1.6.6.8	inconsistency of EC (1.6.6.8 is changed to 1.7.1.7)
1.6.8.1	cysI-J	1.6.8.1, 1.8.1.2	1.8.1.2	1.8.1.2	N.A.	inconsistency of EC
2.3.1.38	fabH	2.3.1.-, 2.3.1.38	2.3.1.41	2.3.1.41	2.3.1.38	inconsistency of EC
2.3.1.40	aas	2.3.1.40, 6.2.1.20	2.3.1.40, 6.2.1.20	2.3.1.40, 6.2.1.20	6.2.1.20	inconsistency of EC
2.4.2.15	deoD	2.4.2.-, 2.4.2.15, 2.4.2.1	2.4.2.1	2.4.2.1	2.4.2.1	inconsistency of EC
2.5.1.1	ispA	2.5.1.1, 2.5.1.10	2.5.1.10	2.5.1.10	2.5.1.1, 2.5.1.10	inconsistency of EC
2.6.-.-	serC	2.6.-.-, 2.6.1.52	2.6.1.52	2.6.1.52	2.6.1.52	inconsistency of EC (incomplete EC)
2.6.1.29	ygjG	2.6.1.29	2.6.1.13	2.6.1.13	2.6.1.13	inconsistency of EC
3.1.3.6	cpdB	3.1.3.6	3.1.4.16	3.1.4.16	N.A.	inconsistency of EC
3.1.3.8	agp	3.1.3.8, 3.1.3.10	3.1.3.10	3.1.3.10	3.1.3.10	inconsistency of EC
3.5.1.44	cheB	3.5.1.-, 3.1.1.-, 3.1.1.61, 3.5.1.44	3.1.1.61	3.1.1.61	N.A.	inconsistency of EC
3.5.4.-	tadA	3.5.4.-	N.A.	3.5.4.-	N.A.	not available in KEGG PATHWAY (incomplete EC)
3.6.1.34	atpA-G	3.6.1.34	3.6.3.14	3.6.3.14	3.6.3.14	inconsistency of EC (3.6.1.34 is changed to 3.6.3.14)
4.1.1.3	eda	4.1.1.3, 4.1.2.14, 4.1.3.16	4.1.2.14, 4.1.3.16	4.1.2.14, 4.1.3.16	4.1.2.14	inconsistency of EC
4.1.2.15	aroF-H	4.1.2.15	2.5.1.54	2.5.1.54	4.1.2.15	inconsistency of EC (4.1.2.15 is changed to 2.5.1.54)
4.1.2.16	kdsA	4.1.2.16	2.5.1.55	2.5.1.55	4.1.2.16	inconsistency of EC (4.1.2.16 is changed to 2.5.1.55)
4.1.2.40	ydjI	N.A.	N.A.	N.A.	N.A.	possible misannotation by GEM System by sequence similarity to
4.1.3.12	leuA	4.1.3.12	2.3.3.13	2.3.3.13	4.1.3.12	inconsistency of EC (4.1.3.12 is changed to 2.3.3.13)
4.1.3.18	ilvB, G-I, M-N	2.2.1.6, 4.1.1.71	2.2.1.6	2.2.1.6	4.1.3.18	inconsistency of EC (4.1.3.18 is changed to 2.2.1.6)
4.1.3.27	trpD	2.4.2.18, 4.1.3.27	2.4.2.18, 4.1.3.27	2.4.2.18, 4.1.3.27	4.1.3.27	KEGG PATHWAY mainly uses 2.4.2.18
4.1.3.31	prpC	2.3.3.1, 4.1.3.31	2.3.3.5	2.3.3.5	4.1.3.31	inconsistency of EC (4.1.3.31 is changed to 2.3.3.1)
4.1.3.9	menD	2.3.3.11, 4.1.1.71	2.5.1.64	2.5.1.64, 4.1.1.71	4.1.1.71	inconsistency of EC (4.1.3.9 is changed to 2.3.3.11)
4.2.1.13	tdcG, yhaP-Q, sdhY	4.2.1.13	4.3.1.17	4.3.1.17	no EC	inconsistency of EC (4.2.1.13 is changed to 4.3.1.17)
4.2.1.14	sdaA-B	4.3.1.17, 4.3.1.19	4.3.1.17	4.3.1.17	no EC	possible misannotation by GEM System (4.2.1.14 is changed to 4
4.2.1.16	tdcB, ilvA	4.3.1.19	4.3.1.19	4.3.1.19	no EC	inconsistency of EC (4.2.1.16 is changed to 4.3.1.19)
4.2.99.11	mgsA	4.2.3.3	4.2.3.3	4.2.3.3	4.2.3.3	inconsistency of EC (4.2.99.11 is changed to 4.2.3.3)
4.2.99.2	thrC	4.2.99.2	4.2.3.1	4.2.3.1	4.2.3.1	inconsistency of EC (4.2.99.2 is changed to 4.2.3.1)
4.2.99.8	cysK, M	2.5.1.47	2.5.1.47	2.5.1.47	4.2.99.8	inconsistency of EC (4.2.99.8 is changed to 2.5.1.47)
4.2.99.9	metB	2.5.1.48	2.5.1.48	2.5.1.48	4.2.99.9	inconsistency of EC (4.2.99.9 is changed to 2.5.1.48)
4.3.1.8	hemC	2.5.1.61	2.5.1.61	2.5.1.61	4.3.1.8	inconsistency of EC (4.3.1.8 is changed to 2.5.1.61)
4.3.99.1	cynS	4.3.99.1	4.2.1.104	4.2.1.104	no EC	inconsistency of EC (4.3.99.1 is changed to 4.2.1.104)
4.6.1.3	aroB	4.6.1.3	4.2.3.4	4.2.3.4	N.A.	inconsistency of EC (4.6.1.3 is changed to 4.2.3.4)
4.6.1.4	aroC	4.6.1.4	4.2.3.5	4.2.3.5	4.2.3.5	inconsistency of EC (4.6.1.4 is changed to 4.2.3.5)
4.99.1.-	cysG	4.99.1.4, 1.3.1.76, 2.1.1.107	4.99.1.4, 1.3.1.76, 2.1.1.107	4.99.1.4, 1.3.1.76, 2.1.1.107	2.1.1.107	inconsistency of EC (incomplete EC)
5.3.1.10	nagB	3.5.99.6	3.5.99.6	left3.5.99.6	3.5.99.6	inconsistency of EC (5.3.1.10 is changed to3.5.99.6)
5.3.1.3	fucI	5.3.1.3, 5.3.1.25	5.3.1.3, 5.3.1.-	5.3.1.25	5.3.1.25	inconsistency of EC
6.3.1.5	yhjG	N.A.	N.A.	N.A.	N.A.	possible misannotation by GEM System by sequence similarity to nadE gene
6.3.2.15	murF	6.3.2.15	6.3.2.10	6.3.2.10	6.3.2.15	inconsistency of EC (6.3.2.15 is changed to 6.3.2.10)
6.3.4.1	guaA	6.3.4.1, 6.3.5.2	6.3.4.1, 6.3.5.2	6.3.5.2	6.3.5.2	KEGG PATHWAY mainly uses 6.3.5.2
